# Why bortezomib cannot go with ‘green’?

**DOI:** 10.7497/j.issn.2095-3941.2013.04.004

**Published:** 2013-12

**Authors:** Li Jia, Feng-Ting Liu

**Affiliations:** 1 Center for Hemato-Oncology, Barts Cancer Institute, St Bartholomew’s Hospital, Barts Health NHS Trust, Queen Mary University of London, London E1 4NS, UK; 2 Division of Hemato-Oncology, St Bartholomew’s Hospital, Barts Health NHS Trust, Queen Mary University of London, London E1 4NS, UK

**Keywords:** Bortezomib, flavonoids, polyphenols, myeloma

## Abstract

Eat more ‘green’ or eat ‘five a day’ is one of the most important healthy lifestyle behaviours in the 21 century. Aiming to fight cancer effectively, more than half patients use vitamins or herbs concurrently with conventional anticancer treatment. Flavonoids or polyphenols existing in vegetables, fruits and green tea are common plant pigments with antioxidant properties and considered acting as cancer preventing or anti-cancer agents. Recently it was found that some flavonoids and vitamin C in diet or supplements have antagonistic effect with the anti-cancer drug bortezomib. Bortezomib is a specific inhibitor for proteasome and is currently used for treatment of relapsed and refractory multiple myeloma. Despite its successful rates in treating multiple myeloma and other solid tumors, it is unable to kill leukemic cells in the blood. It was recently revealed that some flavonoids and vitamin C present in green leaves and green teas in the blood can neutralize bortezomib by directly interaction between two chemicals. Here we summarize why dietary flavonoids should be avoided in patients who take bortezomib as chemotherapeutic drug.

## Introduction

Bortezomib (codenamed as PS-341 and marketed as Velcade by Millennium Pharmaceuticals) was developed as a potent, specific proteasome inhibitor for the treatment of relapsed and refractory multiple myeloma^1^ and currently it is still one of the most effective drugs currently available for treating multiple myeloma. While bortezomib alone achieved a 40% response rate (RR), the RR was further improved to 88% in combination with dexamethasone^2^. However, only 4 of 15 patients with acute leukemia showed a decrease in blast count^3^. Bortezomib has also shown poor efficacy in the treatment of chronic lymphocytic leukemia (CLL), despite potent *in vitro* activity^4^^,^^5^. Recently, it was found that some of dietary flavonoids and vitamin C have antagonistic interaction with bortezomib which affects the anti-cancer property of this drug^6^^-^^9^. According to that 77% of patients use vitamins or herbs concurrently with conventional anticancer treatment^10^, here we review current knowledge concerning how dietary intake could counteract chemotherapy with bortezomib.

## Chemical structure and anti-cancer properties of bortezomib

**Figure 1:**
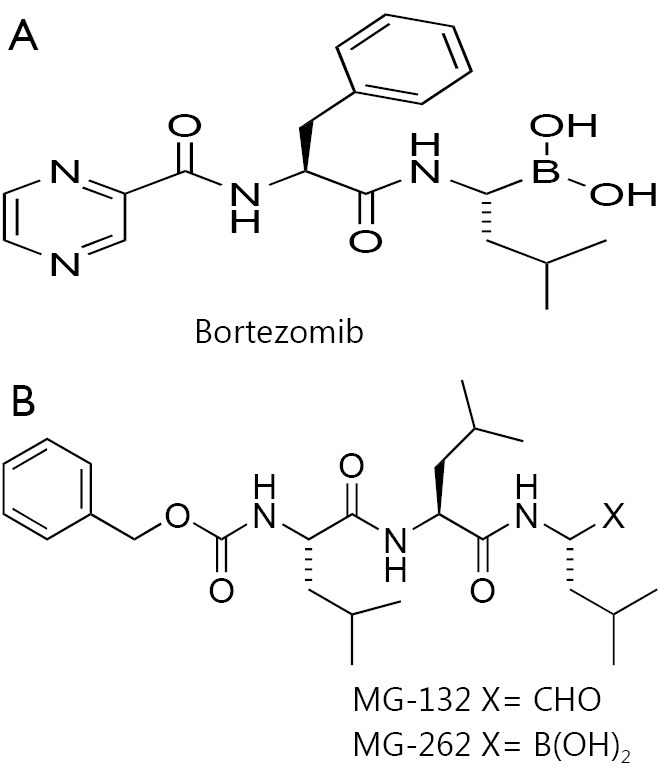
Basic chemical structures of bortezomib (A) and MG-132 or MG-262 (B).

Bortezomib is a modified dipeptidyl boronic acid. The product is provided as a mannitol boronic ester which, in reconstituted form, consists of the mannitol ester in equilibrium with its hydrolysis product, the monomeric boronic acid (**Figure 1**). One of the first proteasome inhibitors synthesized was MG-132, a peptide aldehyde based on calpain inhibitor I^11^. However, MG-132 was found to be nonselective because it inhibits other enzymes. Using a boronic acid instead of an aldehyde circumvents theses hortcomings and provides a measure of selective proteasome inhibition relative to many other serine proteases^2^^,^^12^.

Previous studies suggested that proteasome inhibition by bortezomib kills multiple myeloma cells via blocking inducible I-κB degradation and consequently NF-κB activation implicated as one of the mechanisms of tumor cell resistance to apoptosis^1^^,^^13^^,^^14^. It induces cell cycle arrest and apoptosis in small cancer cells by preventing degradation of p21/waf1, a cyclin-dependent kinase inhibitor 1, and p53^15^. *In vitro* experiment demonstrated that bortezomib also prevents degradation of Bax, a short-lived pro-apoptotic protein, in CLL and diffuse large B-cell lymphoma (DLBCL) cells^16^. Malignant cells can resist by failing to accumulate pro-apoptotic proteins after bortezomib treatment, and/or increase the levels of anti-apoptotic proteins, inducing autophagy to clear up damaged proteins^17^.

## Chemical structure and classification of flavonoids

**Figure 2:**
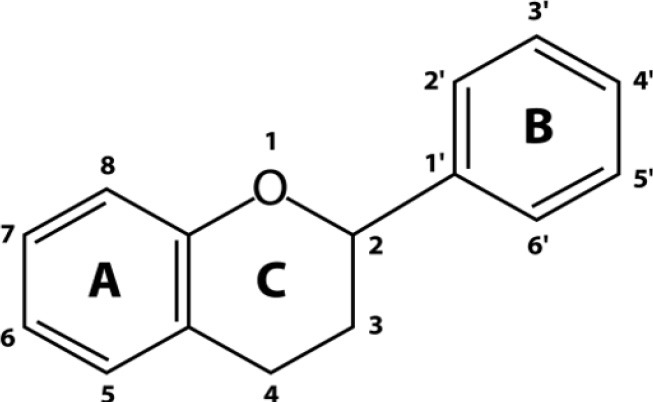
Basic chemical structure of flavonoids.

**Figure 3:**
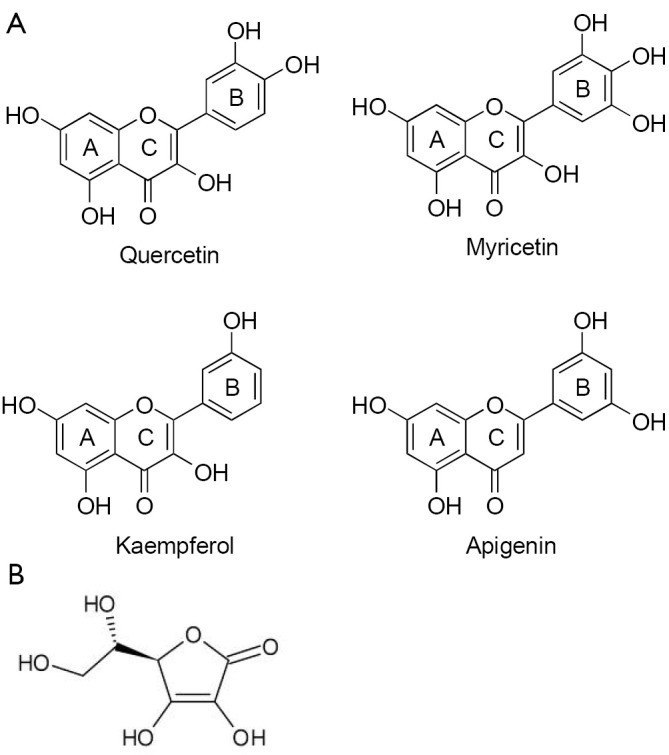
A. Chemical structure of quercetin, myricetin, kaempferol, and apigenin. The B-rings of quercetin and myricetin are catechol and pyrogallo respectively. B. Chemical structure of vitamin C or ascorbic acid.

Flavonoids are biologically active polyphenolic compounds with various health benefits, ubiquitously found in fruits, vegetables, tea, and wine. Flavonoids are benzo-γ-pyrone derivatives consisting of phenolic and pyrane rings (**Figure 2**) and are classified according to substitutions, including flavonols (e.g., quercetin, kaempferol), flavones (e.g., apigenin, luteolin), flavanones (e.g., hesperidin, naringenin), flavan-3-ols (e.g., catechin, theaflavin, and gallic esters of catechin and theaflavins), anthocyanidins (e.g., pelargonidin, cyanidin), and isoflavones (e.g., genistein, daidzein)^18^^,^^19^. They also have different distribution of hydroxyl groups (-OH) in their B ring. For example, quercetin is a catechol with 2 hydroxyl groups (-OH) on neighbouring carbon atoms of their B rings; and myricetin, a pyrogallol, has 3-OH groups, whereas both apigenin and kaempferol have only one isolated -OH group on the B ring (**Figure 3A**). There are many flavonoids containing either catechol or pyrogallol structures in human food sources, such as green vegetables, green tea and fruits (**Table 1**). Interestingly, vitamin C (L-ascorbic acid), an analogue of catechol, contains a vicinal diol group or two neighbouring hydroxyl groups (**Figure 3B**). The hydroxyl configuration on the B-ring of flavonoids and in the vicinal diol group of vitamin C is the most significant determinant of scavenging of reactive oxidative species (ROS)^18^^,^^20^^,^^21^. Hydroxyl groups on the B-ring donate hydrogen and an electron to hydroxyl, peroxyl, and peroxynitrite radicals, stabilizing them and giving rise to a relatively stable flavonoid radical18. However, the complex formation of the vicinal diol in catechol and vitamin C and its simple derivatives with boron acid in aqueous solution has been well characterized chemically for several decades^8^^,^^20^.

**Table 1:** Dietary flavonoids with catechol and pyrogallol structures

Catechols or pyrocatechols	Nature sources	Pyrogallols	Nature sources
Quercetin	Fruits, vegetables, green tea, red wine	Myricetin	Fruits, vegetables and herbs
Catechin	Green tea	Gallic acid	Tea
Epicatechin (EC)	Green tea	Gallocatechin (GC)	Green tea
Epicatechin gallate (ECG)	Green tea	Epigallocatechin (EGC)	Green tea
Caffeic acid	Coffee, fruits, vegetables and herbs	Epicatechin gallate (ECG)	Green tea
Rutin	Buckwheat	Epigallocatechin gallate (EGCG)	Green tea
Luteolin	Fruits, vegetables	Tannin	Tea, wine, fruits, and chocolates
Cyanidin	Color of fruits	Delphinidin	Color of fruits

## Flavonoids quercetin and myricetin diminish the anti-cancer effects of bortezomib

**Figure 4:**
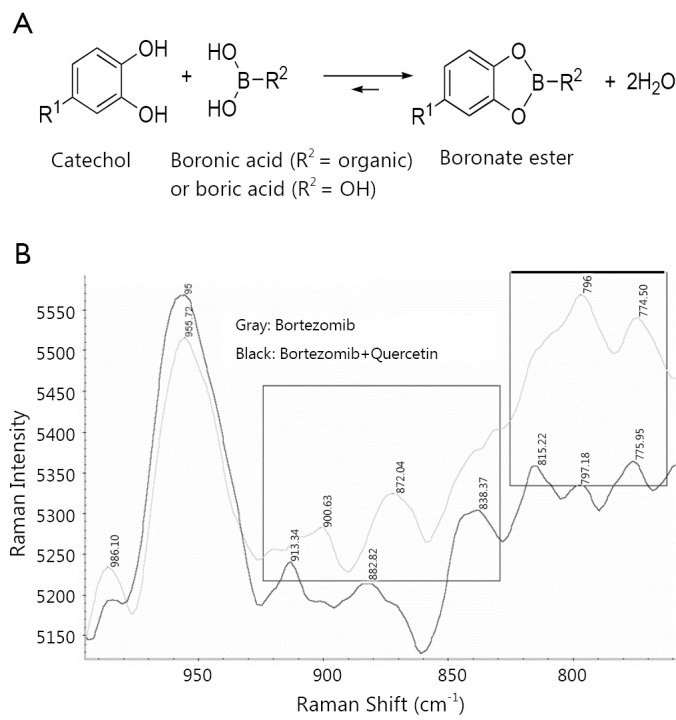
A. Complex formation between catechol derivatives and boronic acid to form boronate ester. B. Detection of chemical reactive between bortezomib and quercetin by Roman Spectrophotometry.

Bortezomib does not kill leukemic cells *in vivo*, despite its potent cytotoxicity *in vitro*^4^. To mimic *in vivo* environment, Liu *et al.*^8^ found that the efficacy of bortezomib dramatically compromised when CLL cells were cultured in 50% fresh human plasma compared to culturing in 10% fetal calf serum, suspecting that unknown antagonistic compounds against bortezomib exist in the blood. Quercetin is one of the abundant flavonol-type flavonoids, commonly found in green leaves of vegetables and fruits. The average daily intake of flavonoids (quercetin, myricetin, kaempferol) and two other flavone-type flavonoids (apigenin and luteolin), was estimated to be 23 mg/day, with quercetin (mean intake, 16 mg/day) as the most consumed of these five flavonoids^22^. Quercetin is rich in the plasma and is extensively plasma-bound, almost exclusively to human serum albumin^23^. The plasma concentration of quercetin is tightly associated with its dietary intake^24^. Quercetin has many functional similarities to bortezomib in the treatment of cancer cells, such as inhibiting proteasome and NF-κB, inducing apoptosis and cell cycle arrest (**Table 2**). However, when treating CLL cells with quercetin and bortezomib simultaneously, the apoptosis-inducing effects of both compounds were vanished completely8. It was thought that quercetin-mediated antagonism on bortezomib was due to inhibition of ROS generation. N-acetylcysteine, a ROS scavenger, failed to inhibit bortezomib-induced cells death, instead enhanced its cytotoxicity. A similar result was produced by another group^7^. The boronic acid group, -B(OH)_2_, which is present in bortezomib, can be expected to form cyclic boronate esters with the catechols and pyrogallols groups (**Figure 4A**), but not with flavonoids such as apigenin and kaempferol in which pairs of adjacent hydroxyl groups are absent (**Figure 3A**). Using Roman spectrophotometry, Liu *et al*., confirmed the directly chemical binding between quercetin and bortezomib (**Figure 4B**).

**Table 2:** Comparison of apoptosis-inducing effects of quercetin and bortezomib

Property	Quercetin	Bortezomib	References
Compound	Flavonoid	Boronic dipeptide	Adams *et al*.^2^; Boulton *et al.*^23^
Proteasome binding site	Inhibition on β5 subunit	Inhibition on β5 subunit	Chen *et al*.^25^
ROS	Inhibition	Generation	Perez-Galan *et al*.^26^
NF-κB	Inhibition	Inhibition	Dias *et al*.^27^
Bax	Activation	Activation	Chen *et al*.^25^; Dias *et al.*^27^
Caspase-3	Activation	Activation	Choi *et al.*^28^; Yu *et al.*^29^
Cell cycle	G_2_/M arrest	G_2_/M arrest	Yang *et al*.^30^; Yin *et al.*^31^
Proliferation	Inhibition	Inhibition	Yang *et al*.^30^; Yin *et al.*^31^

As expected, myricetin showed a similar blocking effect on bortezomib-induced apoptosis as quercetin did but neither apigenin nor kaempferol interfered with bortezomib. The inhibitory effects of plasma on bortezomib cannot be attributed solely to quercetin as its reported peak serum concentration after a supplemental diet is too low^32^. However, there are many dietary flavonoids that have similar structures with quercetin or myricetin (**Table 1**), so the intake of dietary flavonoids may reduce the killing activity of bortezomib on circulating leukemic cells by the formation of a boronate complex.

This study confirmed that the apoptotic effect of MG-262, a boron acid-containing proteasome inhibitor (**Figure 1B**), can also be diminished by quercetin. By contrast, the cytotoxic effect of MG-132 which does not contain boron acid was not affected by quercetin. Importantly, to neutralize flavonoids, inorganic boric acid was added into autologous plasma before treatment with bortezomib. In a non-cytotoxic dose range, boric acid restored apoptosis-inducing activity of bortezomib in a dose-dependent manner^8^.

## Flavonoids in green tea block anti-cancer effect of bortezomib and other boronic acid-based proteasome inhibitor

**Figure 5:**
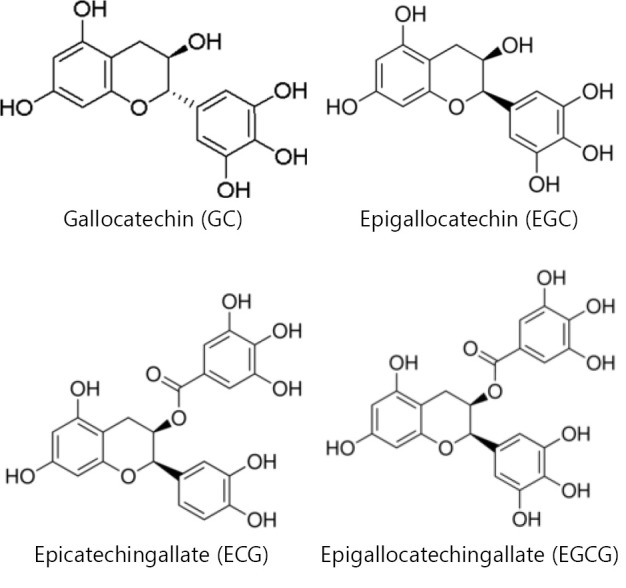
Chemical structures of green tea compounds.

The health benefits and cancer prevention/anti-cancer effects of green tea components have drawn great attention for over two decades due to their anti-oxidant property^33^^-^^36^. The anti-oxidant compounds in green tea, including Gallocatechin (GC), Epigallocatechin (EGC), Epicatechin gallate (ECG), and Epigallocatechin gallate (EGCG), are pyrogallo-based compounds and EGC contains extra catechol ring (**Figure 5**). EGCG, the most bioactive green tea polyphenol has been proposed as a multi-functional chemoprevention and anti-cancer agent^33^^,^^37^^-^^40^.

Golden *et al.*^6^ initially found that 10 µM of EGCG completely blocked 10 nM bortezomib-induced apoptosis in primary and multiple myeloma cell lines, and also in glioblastoma cell lines. EGCG from drug store, called TEA-VIGO, produced similar results on blocking bortezomib as the chemical from Sigma. Other green tea polyphenol components, including EGC, ECG, and EC, all showed blocking effects on bortezomib but required relatively higher concentrations. Complete green tea extract (GTE) also blocked the killing by bortezomib regardless the cytotoxic effect of GTE alone at higher concentration. Similar to the study Liu *et al.*^8^, Golden *et al.*, also tested the antagonistic effect of EGCG on other proteasome inhibitors which either contain boronic acid such as Nelfinavir, PS-1 and MG-132 or without it, such as MG-262 and PX-1X. They found that the protective feature of EGCG is not a general effect toward all proteasome inhibitors, but rather displays selectivity toward those compounds harboring a boronic acid moiety. For *in vivo* study, multiple myeloma cells were implanted subcutaneously into nude mice and, after sizable tumors had formed, the animals received treatment with both bortezomib and/or EGCG. In tumors from animals treated with EGCG (25 to 50 mg/kg) or EGCG plus bortezomib, there is no increase of apoptotic cells compared with tumors from untreated controls. Finally, using ^1^H NMR and ^1^C NMR techniques they confirmed that direct interaction between EGCG and bortezomib lead to formation of a covalent cyclic boronate between these two compounds^6^.

## Vitamin C inhibits anti-cancer effect of bortezomib

Vitamin C or L-ascorbic acid is the most common healthy food supplement for both normal human being and cancer patients. Structurally, it is not a flavonoid, or polyphenol but it contains a vicinal diol (**Figure 3B**) and has a strong anti-oxidant activity. Currently, the effect of vitamin C in cancer prevention and influence on chemotherapeutic drugs remain controversial^41^^-^^43^.

Perrone *et al.*^9^ observed that vitamin C blocks bortezomib-mediated growth inhibition, accumulation of ubiquitinated proteins and inhibition of proteasome activity in multiple myeloma cell lines. This antagonistic effect of vitamin C on bortezomib is limited to peptide boronic class proteasome inhibitors. This group conducted *in vivo* study in xenograft mouse model of human multiple myeloma by treating mice with vitamin C or bortezomib alone or in combination. Bortezomib alone significantly inhibited tumor growth while vitamin C alone showed no effect. Importantly, vitamin C (40 mg/kg/day) completely blocked bortezomib-mediated anti-cancer effect.

## Effects of non-vicinal diol containing naturally occurring compounds on bortezomib

Previous study by Liu *et al.*^8^, demonstrated that non-vicinal diol containing flavonoids such as kaempferol or apignin don’t have antagonistic effect on bortezomib. Flavopiridol is a synthetic flavonoid based on an extract from an Indian plant for the potential treatment of cancer. It was found that flavopiridol (Alvacidib) has synergistic effect on bortezomib-induced killing in chronic myeloid leukemia^44^. A phase I study showed that bortezomib/flavopiridol regimen appears active in patients with relapsed and/or refractory multiple myeloma or non-Hodgkin’s lymphoma^45^. Curcumin is a natural occurring flavonoid extracted from Indian spice turmeric. It was found that curcumin and its analogs enhanced bortezomib-induced apoptosis in multiple myeloma cells^46^^,^^47^. Resveratrol, a nature phenol, is produced by Japanese knotweed, red grapes, berries, and peanuts and is also found at high concentration in red wine. It was reported that resveratrol mediates apoptosis in multiple myeloma cells when used alone or in combination with paclitaxel^48^ or bortezomib^49^. However, a phase II study of SRT501 (a micronized oral formulation of resveratrol) with bortezomib found that SRT501 causes renal failure and minimal efficacy in patients with relapsed/refractory multiple myeloma when used alone or in combination with bortezomib^50^.

## Preclinical *in vivo* studies and controversies

A preclinical study on the antagonistic effect of EGCG or vitamin C in the anti-tumor activity of bortezomib was conducted by Millennium Pharmaceuticals using CWR22 human prostate xenograft tumors^51^. Experiment using multiple myeloma cell line RPMI8226 showed that the concentration of EGCG required for partially inhibiting bortezomib was ≥11 µM, higher than previously reported, e.g., 10 µM for complete inhibition^6^. The plasma concentrations of EGCG were monitored after intravenous (IV) administration of EGCG. Single-agent bortezomib 0.8 mg/kg IV demonstrated a tumor growth inhibition (TGI) of 53.9%-58.9% versus the control group in CWR22 xenograft-bearing mice. EGCG 50 mg/kg IV administered two min prior to bortezomib 0.8 mg/kg IV resulted in no antitumor activity, demonstrating antagonism between EGCG and bortezomib when EGCG levels were >200 µM at the time of bortezomib dosing. Pharmacodynamic studies were conducted to determine whether EGCG showed a concentration-dependent ability to antagonize bortezomib-induced proteasome inhibition in blood or tumor tissue. Four hours after IV administration of bortezomib 0.8 mg/kg, mean 20S proteasome inhibition was 44% in blood and 52% in tumor. This proteasome inhibition was blunted by the combination of EGCG 50 mg/kg IV followed 2 min later by bortezomib 0.8 mg/kg IV, which resulted in only 25% and 33% proteasome inhibition in blood and tumor, respectively, at 4 h post-administration. Golden *et al.*, found that 25 or 50 mg/mL EGCG blocked the apoptosis-inducing effect of 0.5 mg/mL of bortezomib in nude mice. These results indicate partial or complete antagonism of proteasome inhibition by IV dosing of EGCG, which is further reflected in the analysis of downstream pharmacodynamic markers and ultimately in the antagonism of antitumor activity in this combination regimen.

This group also determined the antagonistic effect of vitamin C on bortezomib.Ascorbic acid 40 or 500 mg/kg alone did not exhibit any antitumor activity, while bortezomib 0.8 mg/kg IV alone had significant antitumor activity compared with controls. Surprisingly, they found that ascorbic acid 40 or 500 mg/kg in combination with bortezomib also exhibited significant antitumor activity compared with controls. No antagonism was seen between ascorbic acid and bortezomib in any of the combination groups. This is contradicted with previous study by Perrone *et al.*^9^, which demonstrated that 40 mg/kg ascorbic acid abolished 0.1 mg/kg bortezomib-induced tumor growth inhibition in human multiple myeloma xenograft-bearing mice. It remains unexplained why different xenograft animal models and different ratios of drugs produce different results.

This study^51^ concluded that plasma concentrations of EGCG and ascorbic acid reported in human subjects taking EGCG or vitamin C supplements show no antagonism to the antitumor activity of bortezomib in human prostate tumor fragment xenograft-bearing mice, and therefore there appears no need for patients receiving bortezomib therapy to avoid normal dietary consumption of green tea, vitamin C-containing foods, or EGCG or vitamin C dietary supplements.

## Unanswered questions and perspectives

Although the direct *in vitro* interaction between vicinal diol in flavonoids/vitamin C and boronic acid was detected by several groups^6^^-^^8^, the differential interaction potential in plasma and solid tumor environment was not determined. It is still unclear why bortezomib can kill malignant cells in the tissue and bone marrow but not in the blood. If the anti-cancer property of bortezomib is affected by flavonoids in the blood, how it can reach to the tumor or bone marrow sites unaffected?

Some natural products or antioxidants such as luteolin, ellagic acid, flavonoids, protocatechuic acid, rosmarinic acid, phenethyl caffeate and catechin from vegetables, fruits or herbs have one or more vicinal diol groups. Thus these agents may have the potential to chemically interact with bortezomib and antagonize its activity^20^. Nonetheless, these studies serve as an always timely reminder for healthcare providers of the importance of eliciting a complete history from patients and their families, including concomitant medications and over-the-counter supplements^52^. It is reasonable to suggest to patients that there are potentially negative interactions between proven anticancer therapies and ‘complementary’ therapies. Until we, as researchers and clinicians, have a clear understanding of the potential interactions or lack thereof, we should caution our patients to limit their use to maximize their benefit from treatment^20^.

There are several strategies which may allow further development of proteasome inhibitors for the treatment of leukemia^8^^,^^53^. First, decreasing plasma flavonoid concentrations by dietary manipulation may, if achievable, be of value in enhancing *in vivo* toxicity of bortezomib. Second, the observation that boric acid can compete with the reaction between quercetin and bortezomib raises the possibility that the blocking effect of flavonoids may be neutralized prior to bortezomib treatment. A third option would be to explore the possible therapeutic use of proteasome inhibitors which lack a boronate moiety.
